# Multidisciplinary Oral Rehabilitation of a Severely Compromised Dentition

**DOI:** 10.1155/2020/2429505

**Published:** 2020-02-22

**Authors:** Carlo Maiorana, Dario Andreoni, Paola Polacco, Pier Paolo Poli

**Affiliations:** ^1^Implant Centre for Edentulism and Jawbone Atrophies, Maxillofacial Surgery and Odontostomatology Unit, Fondazione IRCCS Cà Granda Ospedale Maggiore Policlinico, University of Milan, Milan, Italy; ^2^Private Practice, Via Milano 19, 20871 Vimercate, Monza Brianza, Italy

## Abstract

The decision-making process of complex clinical cases should involve multiple specialists to obtain a predictable result on a long-term basis. In view of the above, the present report is aimed at describing the multidisciplinary management of a partially edentulous female patient presenting with a severely compromised residual dentition. To improve function and aesthetics, the treatment combined multiple extractions, temporary rehabilitation with a complete removable denture, guided bone regeneration and implant insertion, soft tissue management, tooth alignment, and restorative dentistry. Thus, several dental branches were embraced during the treatment phases, including oral surgery and implantology, periodontology, orthodontics, and prosthodontics. The involvement of different specialists ensured the achievement of a good result from biological, functional, and aesthetic aspects. The patient was satisfied with the final outcome. In conclusion, to meet the patient's expectations particularly in complex clinical situations, the interdisciplinary approach becomes essential from the early phases in order to identify the ideal treatment plan with the correct time sequence.

## 1. Introduction

The long-lasting failing dentition is a frequent and insidious day-to-day clinical challenge. Common features include heavily restored teeth, incongruous prostheses, residual roots, periapical lesions, abundant deposits of dental calculus, and periodontal disease. The progression of periodontitis is strictly combined with alveolar bone loss, which is mediated by the host immune and inflammatory response to the microbial challenge. For such reason, it is not unusual to observe at the radiographic examination an extensive horizontal bone resorption eventually associated with vertical bony defects at the expense of the alveolar process. Bone remodelling is exacerbated by the use of conventional removable dentures that leads to a pronounced reduction of the mandibular ridge and consequent forward-upward rotation of the mandible. In the lower jaw, this results in the reduction of ridge height and consequent prosthetic retention problems. The lack of fit and stability negatively influence patients' comfort during chewing and speaking.

All these factors are responsible for the low oral health-related quality of life perceived by the patients wearing removable dentures [1]. This explains why a better quality of life can be achieved with implant-retained prosthesis compared to conventional dentures in terms of physical pain and disability, psychological discomfort and disability, and functional limitations [2]. It must be noted that edentulism can lead to a significant functional impairment together with unfavourable aesthetic and psychological changes in patients. Problems include restrictions in diet and limited ability to eat certain foods, speech impairment, loss of support for facial musculature, and decreased vertical dimension. All these drawbacks taken together have made the edentulism to be recognized by the World Health Organization as a physical handicap [3].

All these findings taken together suggest the importance of saving teeth with modest tooth-associated ailments at least during the early stages and usage of dental implants to replace hopeless and/or missing teeth. Therefore, a comprehensive evaluation that implies a multidisciplinary approach is crucial to establish the correct treatment plan based on individual characteristics worked out to meet the patient's expectations on a long-term basis.

In view of the aforesaid, the aim of the present report was to describe the full-mouth rehabilitation of a partially edentulous patient seeking for a fixed rehabilitation adopting a multidisciplinary approach involving the oral surgeon, the orthodontist, and the prosthodontist.

## 2. Case Presentation

The present case was conducted according to the 1964 Helsinki declaration and its later amendments or comparable ethical standards and was reported in compliance with the CARE guidelines (http://www.care-statement.org).

A 67-year-old female patient presented with the chief complaint of tooth mobility, pain while chewing, masticatory limitations, and unsatisfying aesthetic appearance. The patient was healthy, nonsmoking, and with a noncontributory medical history except for a well-controlled hypertension (ASA II according to the American Society of Anesthesiologists physical status classification system).

**Figure 1:**
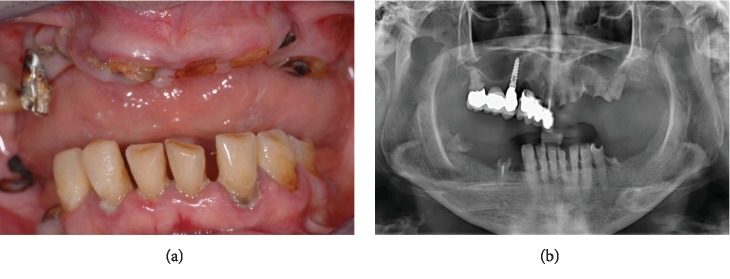
(a) Baseline clinical view of the severely compromised residual dentition; (b) baseline orthopantomograph.

At the clinical intraoral examination, in the maxilla, it was possible to observe a metal-ceramic dental bridge in the frontal right maxilla and an implant-tooth-supported metal-ceramic bridge in the posterior right maxilla. In the mandible, periodontally compromised malpositioned natural elements with calculus deposits were present from the right canine to the first left premolar. In both the jaws, few residual roots were identified. Oedematous and inflamed soft tissues with generalized bleeding on probing and loss of clinical attachment were evident (Figure 1(a)).

The baseline orthopantomograph showed an extensive pneumatization of the maxillary sinuses, with a generalized horizontal bone resorption in both the maxilla and the mandible (Figure 1(b)).

From clinical and radiological findings, both maxillary right metal-ceramic bridges were judged hopeless due to extensive crown structure loss and periodontal disease. Conversely, the maxillary implant was affected by a mild peri-implant mucositis with no loss of supporting bone at the threaded portion, and the residual mandibular teeth could be maintained. Therefore, the preliminary treatment plan consisted of full-mouth extractions of all compromised teeth and residual root stumps and nonsurgical periodontal therapy with manual instruments and piezoelectric ultrasonic unit to eliminate plaque and calculus deposits in the remaining natural elements. Nonsurgical therapy of the implant affected by peri-implant mucositis has been performed with Teflon curettes, ultrasonic scaling with polyether ether ketone- (PEEK-) coated scaler tips, and 0.2% chlorhexidine irrigation. Detailed domiciliary oral health instructions and motivation were given to the patient. A temporary complete removable denture was delivered immediately after the extractions.

After a healing period of 3 months, new clinical and radiological examinations by means of orthopantomograph and cone-beam computed tomography scan were conducted to define possible treatment objectives aimed at restoring function and aesthetic. At this point, two different treatment options were discussed with the patient, namely, a removable or a fixed solution. Following explanation of advantages and disadvantages, the patient refused to wear removable prostheses and preferred fixed implant-supported prosthetic rehabilitations with orthodontic alignment of the mandibular teeth followed by additive composite reconstructions. At the acceptance of the treatment plan, signed informed consent was obtained before starting with the multidisciplinary rehabilitation.

**Figure 2:**
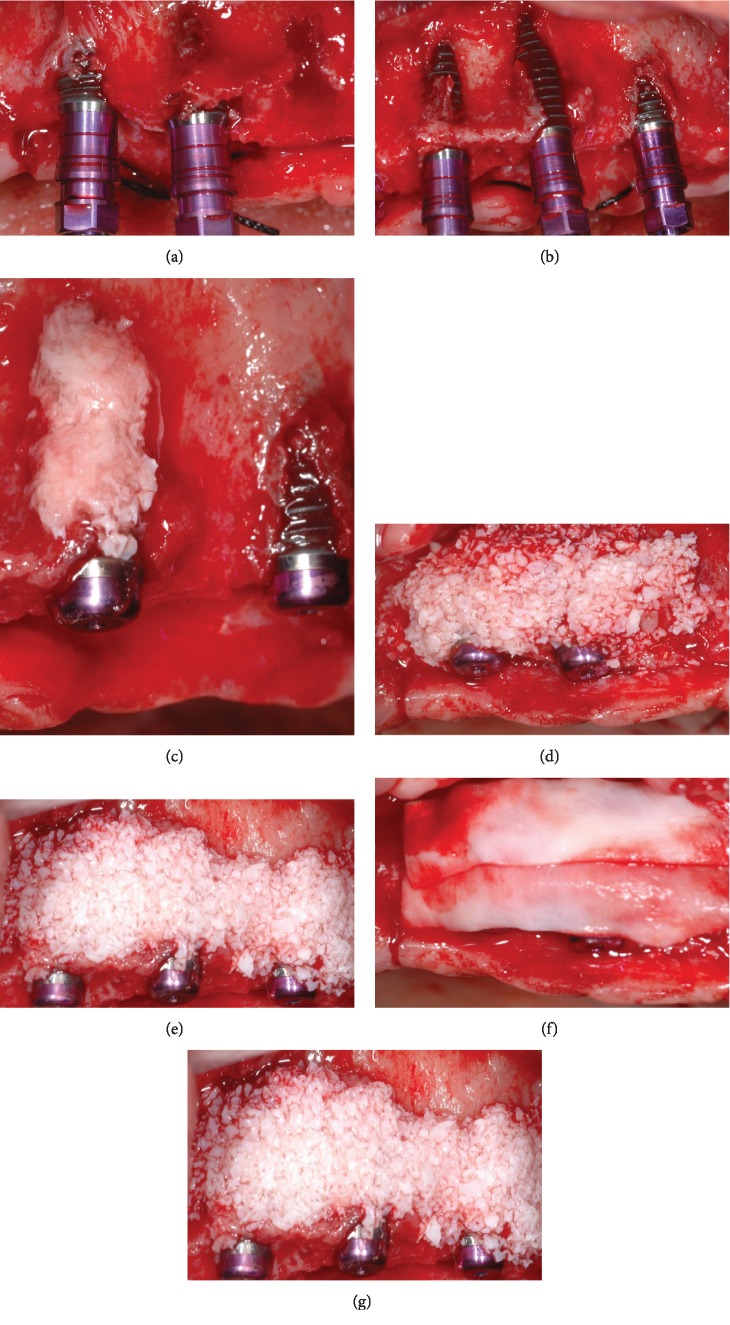
(a, b) Prosthetically guided implant insertion resulting in dehiscences and fenestrations of the buccal bone; (c) autogenous bone particles retrieved from the implant bed preparation in direct contact with the exposed implant threads; (d, e) deproteinized bovine bone particles grafted to overcorrect the bone defect; (f, g) resorbable collagen membranes used with a double-layer technique to cover the graft.

The first step consisted in the insertion of 5 implants (AnyOne®, MegaGen Implant Co. Ltd., Daegu, South Korea) in the maxilla. Both clinical and radiographic evaluations showed a narrow edentulous ridge with significant bone resorption in the buccopalatal dimension. After the elevation of a full-thickness flap, implants were placed according to the manufacturer's instructions with an insertion torque of roughly 30 Ncm. The ideal position of the implants was achieved with the aid of a surgical stent based on the preliminary prosthetic wax-up equipped with a 2 mm diameter guiding hole. Implants were located in correspondence with right and left canines, right and left central incisors, and left second premolar. The prosthetically guided insertion resulted in bone dehiscences and fenestrations of the buccal plate around the implants (Figures 2(a) and 2(b)). Hence, guided bone regeneration (GBR) was performed by grafting autogenous bone chips retrieved from the drilling sequence in direct contact with the exposed implant threads (Figure 2(c)) and deproteinized bovine bone mineral particles (Bio-Oss®, Geistlich Biomaterials, Wolhusen, Switzerland) to overcorrect the bone defect (Figures 2(d) and 2(e)). The graft was then covered with double-layer resorbable collagen membranes (Bio-Gide®, Geistlich Biomaterials, Wolhusen, Switzerland) to create a secluded space and promote undisturbed healing (Figures 2(f) and 2(g)). In the same surgical session, a lateral antrostomy was performed to access the left maxillary sinus. After careful elevation of the Schneiderian membrane, deproteinized bovine bone mineral particles (Bio-Oss®, Geistlich Biomaterials, Wolhusen, Switzerland) were grafted to complete the maxillary sinus floor elevation. This procedure was performed as a preventive measure allowing placement of an additional implant in case the most distal right fixture would have been lost during the subsequent phases. Following horizontal periosteal releasing incisions of the buccal flap, a tension-free first-intention healing was achieved with 5-0 monofilament nylon suture (Ethilon™, Ethicon Inc., Somerville, NJ, USA).

Almost the same surgical procedure was performed after one month in the mandible. A total of 3 implants (AnyOne®, MegaGen Implant Co. Ltd., Daegu, South Korea) were inserted with a torque of approximately 35 Ncm to replace one premolar and one molar in the right side and one molar in the left side. Bone augmentation procedures were unnecessary in this region due to an adequate amount of bone in both horizontal and vertical dimensions.

**Figure 3:**
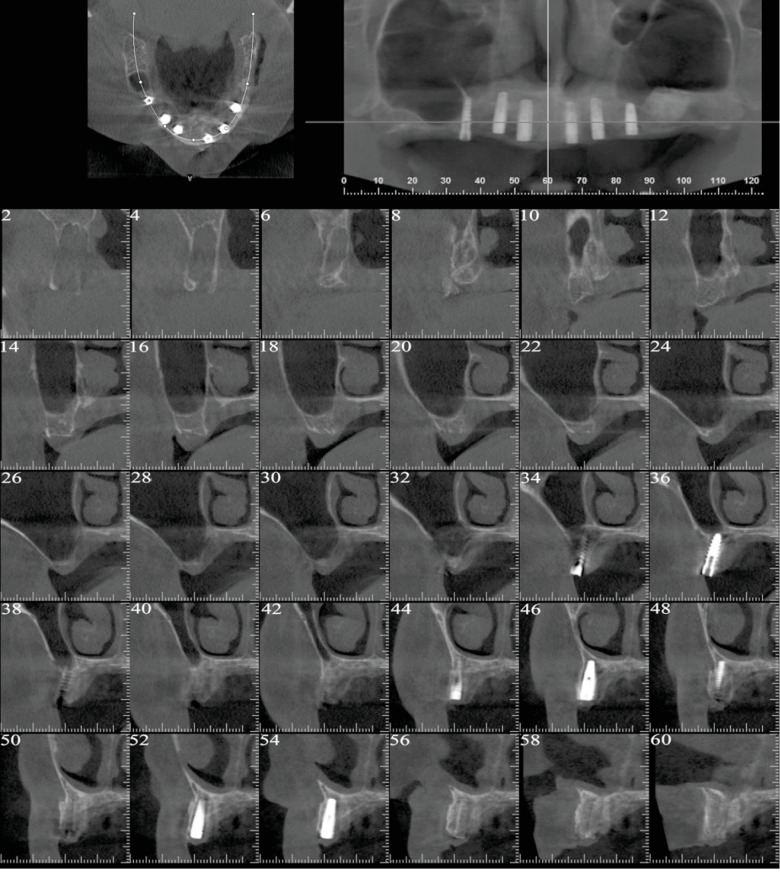
Postoperative tomographic scan showing radiopaque regenerated newly formed bone at the buccal aspect of the implants.

The healing proceeded uneventfully, and the reentry surgery was performed to connect the healing abutments after 6 months in the maxilla and 4 months in the mandible. Before the second stage surgery, a tomographic scan of the maxilla was obtained to evaluate the amount of bone regeneration (Figure 3). In the upper jaw, an apically repositioned flap with the crestal incision slightly displaced toward the palatal aspect was carried out to gain keratinized mucosa at the buccal aspect of the implants. The soft tissues around the implant already present in correspondence with the right second premolar were enhanced by means of an epithelialized free-gingival graft harvested from the homolateral hemipalate. With respect to the mandible, a simple crestal incision was performed to split the keratinized mucosa and uncover the implants.

With regard to the pharmacological management of the surgical interventions, the patient was prescribed antibiotics (amoxicillin clavulanate 875/125 mg tablets) to prevent postoperative surgical site infections, nonsteroidal anti-inflammatory drugs (ibuprofen 600 mg effervescent granules) for pain relief, and 0.2% chlorhexidine gluconate oral rinse to reduce the bacterial load. The patient was also instructed to apply ice packs topically for 3 hours postoperatively.

Following the initial adaptation of the soft tissues, impressions were taken to begin with the prosthetic phases. An implant-supported screw-retained fixed temporary prosthesis was delivered in the maxilla, while implant-supported provisional resin crowns were screwed to the mandibular implants for soft tissue conditioning.

**Figure 4:**
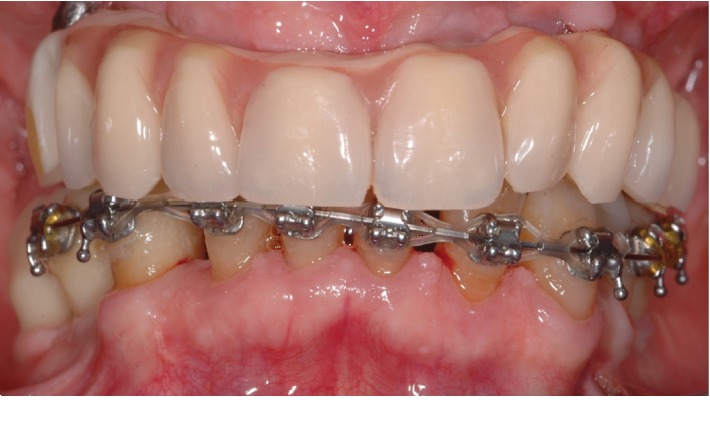
Intraoral clinical view of the interim prostheses in situ during the soft tissue conditioning of peri-implant tissues and orthodontic alignment of the lower teeth.

**Figure 5:**
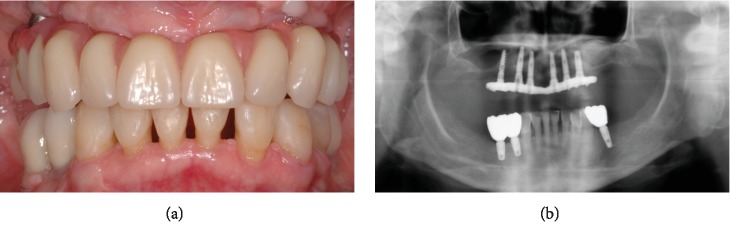
(a) Maxillary and mandibular definitive implant-supported screw-retained fixed dental prostheses associated with additive composite reconstructions of the mandibular teeth; (b) follow-up orthopantomograph performed 2 years after the conclusion of the multidisciplinary treatment.

The interim prostheses were left in situ for approximately 7 to 8 months to allow proper maturation of the peri-implant soft tissues. During this timeframe, the orthodontist proceeded with the alignment of the lower teeth (Figure 4). The final rehabilitation consisted of definitive implant-supported screw-retained fixed dental prostheses associated with additive composite reconstructions of the mandibular teeth performed by the prosthodontist (Figure 5(a)). The main goals of the treatment were achieved restoring stable and functional occlusion and chewing and speech abilities, together with a pleasant aesthetic.

The patient was enrolled in a regular supportive peri-implant/periodontal therapy consisting of supra- and submucosal biofilm removal at the treated implants using titanium or carbon fibre curettes and ultrasonic devices and professional prophylaxis at residual teeth in association with oral hygiene reinforcement. Recall visits were scheduled every 3 months for the first year and twice a year thereafter.

The follow-up orthopantomograph performed after 2 years showed a radiologically healthy situation characterized by stable peri-implant bone levels and no further bone loss around natural teeth compared to the baseline (Figure 5(b)).

## 3. Discussion

Loss of teeth affects the function of the stomatognathic system, particularly if left untreated over an extended period of time. Edentulism not only affects the adjacent teeth that may move undesirably or the opposing teeth that tend to extrude. It has been demonstrated that dimensional ridge resorption inevitably occurs following tooth loss [4, 5]. Advanced resorption of the alveolar bone associated with the physiological pneumatization of the maxillary sinus may preclude proper insertion of dental implants to replace missing teeth. Bone atrophy associated with long-standing malocclusion leads to a decline in masticatory and speech function, compromises the aesthetic appearance, renders the management of oral hygiene more difficult, and becomes a risk factor in the development of periodontal disease.

Such a progressive decay has been well represented by the case reported herein. A partially edentulous patient with atrophic edentulous ridges presented with an extremely compromised residual dentition, generalized chronic periodontitis, impaired function, and unpleasant aesthetic appearance.

The initial aim was to extract the hopeless teeth, bearing in mind that teeth even compromised because of periodontal disease or endodontic problems may have a longevity that surpasses by far that of the average implant [6]. Thus, any nonextractive treatment that is aimed primarily at dental element preservation has been considered before tooth extractions.

At the same time, periodontal disease had to be controlled on the remaining dentition before implant insertion. This is of paramount importance, since patients diagnosed or with a history of periodontitis have a higher risk of developing peri-implantitis than healthy subjects [7]. In patients with a history of periodontitis, putative periodontal pathogens appear to predominate in the microbiome of diseased peri-implant tissues, which confirms previous observations of periodontitis-associated species in deepened pockets around implants [8]. To strengthen the association between periodontitis and peri-implantitis, culture techniques have suggested that the microflora present in the oral cavity before implantation determines the composition of the newly establishing microflora on implants [9]. Given that natural teeth may act as a reservoir for pathogens colonizing implants, and patients with a history of periodontitis are at higher risk to develop peri-implantitis, an initial nonsurgical periodontal therapy has been performed before implant insertion. In the present case, active nonsurgical periodontal therapy consisted of meticulous supra- and subgingival scaling and root planing of all teeth involved by means of ultrasonic and hand instruments. According to the present case, in patients with adult periodontitis, active nonsurgical periodontal therapy yielded effective results, particularly in the case of single-rooted front teeth and premolars [10]. As a result, the patient treated herein showed no residual bleeding on probing and probing pocket depths < 5 mm around the remaining teeth before implantation. The combination of amoxicillin-metronidazole has demonstrated synergic effects, and it has been recommended as an adjuvant to nonsurgical periodontal treatment in the management of periodontitis. However, adjunctive systemic antibiotics shall only be considered according to the severity and extent of the disease. In compliance with recent recommendations, in the present case, antibiotics were not prescribed because the patient is aged >56 years and showed probing depths > 4 mm in less than 35% of sites [11].

At this point, implant insertion was needed to replace the missing teeth according to the patient's wishes. It is needless to say that an adequate amount of bone volume is mandatory at the recipient site to obtain predictable functional and aesthetic results. In reconstructed atrophic ridges, dental implant rehabilitations provided encouraging survival rates on the long term [12, 13]. Amongst the techniques used to augment resorbed edentulous sites, implants placed in association with GBR procedures presented safe and predictable long-term clinical results [14]. In brief, as performed in the present clinical case, bone regeneration is achieved by promoting the repopulation of solely slower-growing osteoprogenitor cells into the bone defect. Barrier membranes are therefore used to prevent the migration of rapidly proliferating epithelium and connective tissue cells and other nonosteogenic tissues into the site to be augmented. By exploiting the barrier function of the membrane, undisturbed new bone formation can occur inside a secluded compartment starting from the surface of the pristine bone [15, 16]. According to modern concepts, implants should be inserted in the ideal position in bone augmented so as to closely match and fully support the rehabilitation plan. In this way, the original concept of restoration-driven implant placement is combined with modern prosthetically guided bone regeneration aimed at following the prosthetic needs in order to achieve the anticipated treatment outcome. In the present case, due to the centripetal and apical resorption pattern of the maxillary alveolar bone, the maxillary osseous base was internal to the tooth position. For such reason, in the present case, GBR procedures were performed in conjunction with prosthetically driven implant insertion to augment horizontally the supporting bone on the buccal aspect of the implants. The outcome of the horizontal augmentation was clearly visible in the 6-month computed tomography scan showing mineralized newly formed bone buccal to the implants.

The management of the soft tissues is a key issue that should not be underestimated. Although there is no consensus available that identifies the absence of keratinized mucosa as a risk factor for peri-implantitis [17], emerging evidence suggests that a reduced width of keratinized tissue around dental implants might be considered a risk indicator for severity of peri-implant mucositis [18]. The fact that peri-implant mucositis is considered to be the precursor of peri-implantitis supports the finding that the absence of keratinized tissue is strongly associated with peri-implant lesions [19]. For such reasons, in the present case, soft tissue augmentation procedures have been performed to improve the quality of peri-implant soft tissues and to increase the width of keratinized mucosa around the implants. It is worth mentioning that both apically positioned flap and autogenous soft tissue grafts result in more favourable peri-implant health in terms of gingival index values and marginal bone level stability [20].

The present report emphasizes the importance of a multidisciplinary approach required for comprehensive care and optimal posttreatment functional and aesthetic outcomes. A thorough knowledge of the relationship between the bone morphology, the periodontal and peri-implant soft tissues, and the three-dimensional position of teeth and implants, with the aid of restorative and prosthetic dentistry, is essential to obtain a good result based on an individualized patient-centred treatment plan.
